# Renal Denervation in Hypertensive Patients: Back to Anatomy?

**DOI:** 10.1161/HYPERTENSIONAHA.120.15834

**Published:** 2020-08-31

**Authors:** Alexandre Persu, Frédéric Maes, Jean Renkin, Atul Pathak

**Affiliations:** 1 From the Division of Cardiology, Cliniques Universitaires Saint-Luc (A. Persu, F.M., J.R.), Université catholique de Louvain, Brussels, Belgium; 2 Pole of Cardiovascular Research, Institut de Recherche Expérimentale et Clinique (A. Persu, F.M.), Université catholique de Louvain, Brussels, Belgium; 3 Department of Cardiovascular Medicine, Centre Hospitalier Princesse Grace, Monaco (A. Pathak); 4 Clinique Pasteur-ESH Hypertension Excellence Center, INSERM 1048, Toulouse, France (A. Pathak).

**See related article, pp 1240–1246**

A decade ago, when endovascular renal sympathetic denervation (RDN) using radiofrequency energy was first proposed as a treatment of resistant hypertension,^1^ the strategy of renal nerve ablation, by that time using the mono-electrode Simplicity catheter, was almost purely empirical, based on porcine model. It was advised to distribute a few ablation points along a spiral path to increase RDN efficacy, while avoiding both circumferential nerve destruction and side branch approach, considered as potentially dangerous. At most, it was whispered to target the upper proximal quadrant of the renal artery where the density of nerve fibers was purportedly the highest. Besides a few dissenting voices,^2^ expectations were huge, and some were thinking RDN would provide a simple solution to the overwhelmingly complex problem of hypertension.

SYMPLICITY HTN-3^3^ was expected to be the cherry on the cake, providing definitive evidence of the efficacy of RDN. As we all know this was not the case, for a number of reasons whose detailed analysis is beyond the scope of this Editorial. This failure was a signal for both investigators and companies that something had gone wrong in the development of RDN or that renal sympathetic nerves do not mediate the entire effect of sympathetic outflow in modulating blood pressure. Methodological and technical issues came to the forefront. Among many others, the optimal strategy to decrease renal sympathetic flow in an efficient and reproducible way was the object of intense debate. The negative results of SYMPLICITY HTN-3^3^ were partly due, it was thought, to an insufficient number of ablation points in most patients, and a post hoc propensity score–adjusted analysis suggested a correlation between the number of ablation points and blood pressure decrease after RDN.^4^ Second, it was advocated that distal instead of proximal nerve fibers should be targeted in priority.^5^ Even though distal innervation was less dense than in the proximal part of the artery, distal nerves were indeed closer to the lumen and so more accessible to destruction.^6^ Finally, compared with RDN of main renal arteries alone, combined ablation of main renal arteries and branches resulted in improved blood pressure–lowering efficacy of RDN.^7^

The development of multielectrode radiofrequency and new-generation ultrasound renal nerve ablation systems has improved the reproducibility and completeness of RDN, and in the context of rigorously designed sham-controlled trials, the blood pressure–lowering effect of RDN is now consistently demonstrated.^8^ Denervation of small branches and accessory arteries remains challenging, but the issue is being partly solved with the design of smaller diameter catheters. Still, despite these technical improvements, the overall benefit of RDN remains modest, roughly equivalent to that of one antihypertensive drug, reliable noninvasive predictors of blood pressure response to RDN have not been consistently identified,^8^ and the potential impact of anatomic variability on the results of RDN remains to be properly addressed.

In this context, the anatomic study of Garcia Touchard et al^9^ published in this issue of *Hypertension* could open new pathways to improve selection of patients and results of RDN. The authors meticulously dissected renal nerves of 60 kidneys from 30 randomly selected cadavers and carefully described their origin and trajectory. This painstaking task proved rewarding, as the authors’ findings challenge a number of preconceptions on renal nerve anatomy. In summary (Figure), (1) the classical basket-type arrangement at the origin of main renal arteries was found in only 17% of cases, and in fact in the majority of cases (57%), such a renal plexus simply did not exist; (2) instead, or in addition, renal innervation originated from large nerve bundles that formed the preaortic ganglia and splanchnic nerves; (3) in more than half of cases (73% on the right and 53% on the left), renal nerves bypassed the main renal artery and joined directly bifurcations or arterial branches. The presence of such late arriving nerves (LAN) and very late arriving nerves (VLAN) strongly correlated with the presence of extra-hilar arterial branching and polar arteries. In contrast, LAN and VLAN were not observed in case of accessory or multiple renal arteries. Finally, the ratio between the length of the main renal artery and the distance from ostium to renal hilum was negatively associated with the presence of LAN/VLAN. In other words, for a fixed ostium-hilum distance, the shortest the renal artery before bifurcation, the highest the probability of LAN/VLAN.

**Figure.**
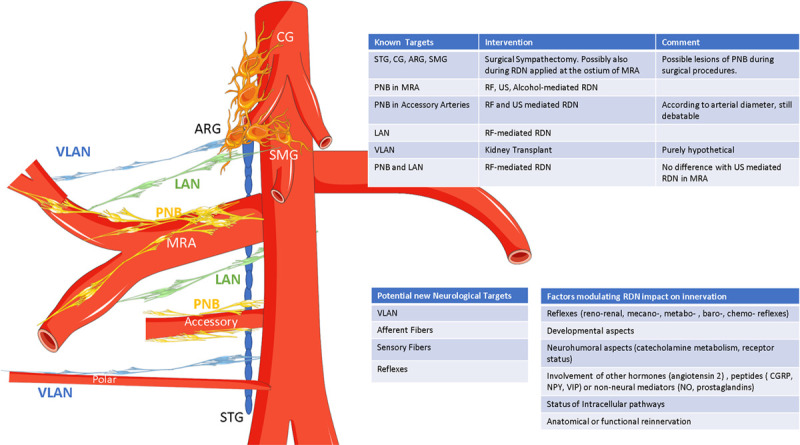
**Anatomy and function of renal sympathetic system: relevant aspects for renal denervation. Left**, abdominal sympathetic ganglia and renal sympathetic innervation. The right celiac ganglia (CG), right aorticorenal ganglia (ARG), and right superior mesenteric ganglia (SMG) are often fused together, partly also with the sympathetic trunk ganglia (STG). The main renal artery (MRA) is innervated by periadventitial nerve bundle (PNB), the bifurcation by late arriving nerves (LAN) and distal branches by very late arriving nerves (VLAN). Accessory arteries are innervated by PNB and polar arteries by VLAN. Left MRA anatomy and innervation might be different from right MRA. **Right**, sympathetic targets for various types of interventions and factors likely to modulate the impact of renal denervation (RDN) on renal innervation, beyond anatomy. CGRP indicates calcitonin gene-related peptide; NPY, neuropeptide Y; RF, radiofrequency; US, ultrasound; and VIP, vasointestinal peptide.

These results support the concept that ablation of renal fibers associated with arterial branches and polar arteries is of importance,^7^ particularly in patients without accessory or multiple renal arteries. If the latter is impossible to achieve, RDN may not be the preferred treatment option for these patients. From a research-and-development perspective, and in view of the high prevalence of LAN and VLAN, the work of Garcia Touchard et al^9^ also highlights the critical importance to develop catheters small enough to reach such branches. Nevertheless, even this may not provide a technical solution for all cases, as some of nerves really land very distally, that is, in segmental branches.^9^ Another way to circumvent the problem could be to use ethanol-based RDN,^10^ as ethanol diffusion may allow reaching LAN and VLAN, thus allowing a more complete denervation of distal arterial branches. This theoretical advantage nevertheless remains to be demonstrated.

Still, the work of Garcia Touchard et al^9^ needs further validation. First, though it is unlikely that many research groups will be able and willing to replicate such a tedious study, it would be of interest to see whether the findings of the Spanish group^9^ apply indifferently to subjects from different ethnic origins, with or without resistant hypertension, cardiovascular, and renal comorbidities. Second, it may be of interest to look retrospectively for a correlation between the ratio length main renal artery/length ostium-hilum, as a proxy for the presence of LAN/VLAN and blood pressure decrease after RDN in existing clinical studies. If such a correlation is found, incorporation of this ratio in upcoming RDN trials would deserve consideration to check prospectively its value as a predictor of blood pressure response to RDN.

Finally, anatomy does not necessarily reflect physiology and pathophysiology of the sympathetic nervous system. Locally, overactivity of the sympathetic nervous system is also related to (1) synthesis of catecholamines (ie, tyrosine pathway and polymorphism of enzymes), (2) release of catecholamines at presynaptic level but also regulation of release through presynaptic receptors, (3) use and metabolism of catecholamines in the synaptic cleft, and finally, (4) postsynaptic binding of catecholamines. The brain-kidney sympathetic nervous system pathways might be overactive irrespective of the number or anatomic distribution of renal nerves (Figure). Most probably, combining anatomy and function as done with functional magnetic resonance imaging in the brain could be a valuable option to further improve tailoring of RDN.

Will more refined selection of candidates based on renal nerves anatomy combined with technical improvement allow achieving better results and contribute to make of RDN a reasonable alternative to antihypertensive drug treatment, beyond the subset of patients with resistant hypertension? This remains to be demonstrated. Anyhow, the recent past of RDN has—expectedly—shown that careful step-by-step optimization is preferable to premature gold rush.

Another remarkable lesson of the study of Garcia Touchard et al^9^ is that we have still to learn from anatomy and that dissection is not obsolete and should remain part of the medical curriculum. Else, someday, despite all sophisticated computer-based medical imaging, we may be unable to address important medical and research questions.

## Sources of Funding

None.

## Disclosures

A. Pathak reports having received research grants from CVRx, Recor, Medtronic, and Ablative Solution and travel grants and speaker fees from CVRx, Recor, Medtronic, and Ablative Solution. A. Persu reports having received travel grants from Ablative Solution. The other authors report no conflicts.
